# The synergistic and enhancive effects of IL-6 and M-CSF to expand and differentiate functional dendritic cells from human monocytes under serum-free condition

**DOI:** 10.1186/s13036-023-00325-z

**Published:** 2023-01-26

**Authors:** Chao-Ling Yao, Tsung-Yu Tseng

**Affiliations:** Department of Chemical Engineering, National Cheng Kung University, No. 1, University Road, Tainan, 70101 Taiwan

**Keywords:** Dendritic cell, Serum-free, IL-6, M-CSF, Immunotherapy

## Abstract

**Background:**

Dendritic cells (DCs) are differentiated from monocytes, and have a strong ability to perform phagocytosis, present antigens and activate T cell immune response. Therefore, DCs are one of the key factors in fighting cancer in immunotherapy, and it is an important issue to develop a serum-free system for DC differentiation and expansion in vitro for clinical application.

**Results:**

In this study, IL-6 and M-CSF were determined and a concentration combination of cytokines was optimized to develop an optimal DC serum-free differentiation medium (SF-DC Optimal) that can effectively differentiate CD14^+^ monocytes into CD40^+^CD209^+^ DCs. After differentiation, the morphology, growth kinetics, surface antigen expression, phagocytosis ability, cytokine secretion, mixed lymphocyte reaction and stimulation for maturation of the differentiated DCs were checked and confirmed. Importantly, this research is the first report finding that the addition an extra low concentration of IL-6 and M-CSF exhibited a synergistic effect with GM-CSF and IL-4 to generate higher numbers and more fully functional DCs than the addition of GM-CSF and IL-4 only under serum-free condition.

**Conclusion:**

A large number of functional DCs can be generated by using SF-DC Optimal medium and provide an alternative source of DCs for related basic research and clinical applications.

## Background

Therapy that affects the immune system to cure disease has been summarized as immune therapy. The low invasion rate, side effects, and recurrence rates are advantages of immune therapy, which provides a new method to cure disease and cancer [1–3]. Antigen-presenting cell (APC) injection is one of the immunotherapies used to activate the immune response. APCs can capture antigens, process antigens and present antigens to T cells and can enhance the immune and cytolytic ability against pathogen invasion and cancer occurrence. Dendritic cells (DCs) have been proven to be the most powerful APCs and have played an important role in immunotherapy in recent years [4, 5]. In humans, DCs mainly differentiate from CD14^+^ monocytes, express CD1a, CD11c, CD40 and CD209, and release interleukin (IL) − 1β, IL-8, IL − 10 and IL − 12 to communicate with other cells in immune system. After stimulation by the uptake of antigens (such as short nucleic acids and lipopolysaccharide (LPS)) or cytokines (such as tumor necrosis factor-α (TNF-α)), CD80, CD83 and CD86 expressions on DCs are enhanced, and the ability of DCs to stimulate T-cell proliferation is enhanced [6–10].

In practice, the major limitation of DC immunotherapy is an insufficient number of DCs and 10 [8] DCs per treatment is required [11–16]. Currently, the common method to differentiate DCs from monocytes is to add high concentrations of IL-4 and granulocyte-macrophage colony-stimulating factor (GM-CSF) under serum-containing culture conditions [17–20]. Although serum contains many growth factors and nutrition that are commonly added to cell culture medium to support cell growth and differentiation, serum also might contain unclear pathogens that is not suitable to produce cells for clinical application. Especially for DC culture, the uptake of allogeneic or xenogeneic proteins from serum into DCs may affect the growth, characteristics and biological functions of DCs. Therefore, the development of a monocyte-derived DC differentiation culture system under serum-free conditions is an urgent and important issue for studies of DC differentiation and immunotherapy.

In a previous study, we determined thirteen serum substitutes to replace the role of serum for DC differentiation from monocytes in the presence of GM-CSF and IL-4 (named SF-DC Control medium) [21]. After 5 days of differentiation in SF-DC Control medium, one DC was generated from every nine CD14^+^ monocytes on average. We noted that adding only GM-CSF and IL-4 may not be enough for DC differentiation under serum-free conditions. Therefore, the aim of this study was to select more effective cytokines and optimize concentration of cytokines under serum-free condition (SF-DC Optimal medium) to generate a large number of functional DCs from monocytes. In this study, the effective cytokines in the SF-DC Optimal medium were screened and optimized by factorial design and the steepest ascent method. In addition to GM-CSF and IL-4, our results demonstrated that IL-6 and macrophage colony-stimulating factor (M-CSF) are also necessary for DC differentiation under serum-free condition. After the SF-DC Optimal medium was developed, the characteristics and functionalities of differentiated DCs were analyzed and were furtherly compared with DCs differentiated in SF-DC Control medium with or without serum. Finally, our results demonstrated that IL-6 and M-CSF exhibit synergistic and enhancive effects with GM-CSF and IL-4 on DC differentiation, and SF-DC Optimal differentiation medium can efficiently generate a large number of functional DCs for basic research and clinical application.

## Results

### Cytokine screening for DC differentiation from CD14^+^ monocyte- factorial design

In our previous study, we developed an SF-DC Control medium comprising RPMI 1640, two cytokines (GM-CSF and IL-4) and thirteen serum substitutes for DC differentiation from human monocytes [21]. To explore more cytokines that were beneficial for DC differentiation under serum-free condition, fifteen cytokines (IL − 1β, IL-2, IL-3, IL-6, IL-7, IL − 12p70, IL-15, IL-16, IL − 17A, Flt3-ligand, SCF, HGF, TGF-β, IFN-β and M-CSF) were selected to test the effects based on extensive review and previous experience [22–32]. Each cytokine was added individually to the SF-DC Control medium. In our preliminary testing and results, IL − 1β, IL-6, IL-7 and M-CSF showed significant effects on the differentiated CD40^+^CD209^+^ DC number or the percentage of CD40^+^CD209^+^ DCs in total cells, and other cytokines did not show a positive effect on DC generation from monocytes (data not shown). Subsequently, a 2^4^ full factorial matrix (Table 1) was adopted to identify the individual and interactive effects of IL − 1β, IL-6, IL-7 and M-CSF on the total cell number, percentage of CD40^+^CD209^+^ DCs in the total cells and CD40^+^CD209^+^ DC number after 5 days of differentiation in SF-DC Control medium (containing GM-SCF and IL-4), and the first-order model was regressed and is listed below.

1 $$\textrm{Total}\ \textrm{cells}/1.5\ \textrm{mL}\ \left(\times {10}^4\right)=4.24-0.63{x}_1+0.11{x}_2-0.19{x}_3-0.23{x}_1{x}_2-0.03{x}_1{x}_3-0.14{x}_1{x}_4-0.06{x}_2{x}_3-0.23{x}_2{x}_4+0.13{x}_1{x}_2{x}_3+0.39{x}_1{x}_2{x}_4+0.19{x}_1{x}_3{x}_4-0.23{x}_2{x}_3{x}_4-0.04{x}_1{x}_2{x}_3{x}_4$$

2 $$\textrm{Percentage}\ \textrm{of}\ \textrm{CD}4{0}^{+}\textrm{CD}20{9}^{+}\ \textrm{DCs}\ \textrm{in}\ \textrm{the}\ \textrm{total}\ \textrm{cells}\ \left(\%\right)=67.26+0.36{x}_1+1.39{x}_2+0.51{x}_3+5.54{x}_4-2.11{x}_1{x}_2-7.86.{x}_1{x}_4-0.16{x}_2{x}_3-3.94{x}_2{x}_4-0.93{x}_3{x}_4-0.48{x}_1{x}_2{x}_3+4.01{x}_1{x}_2{x}_4+0.14{x}_1{x}_3{x}_4+0.23{x}_2{x}_3{x}_4+0.31{x}_1{x}_2{x}_3{x}_4$$

**Table 1:** Matrix of the 2^4^ full factorial design and experimental results of cytokine screening for CD40^+^CD209^+^ cells differentiated from CD14^+^ monocytes in the SF-DC Control medium^a,b^

Trial	IL-1β	IL-6	IL-7	M-CSF	Total cells (×10^**4**^ cells)^**c**^	CD40^**+**^CD209^**+**^ (%)^**c**^	CD40^**+**^CD209^**+**^ cells (×10^**4**^ cells)^**c**^
1	-1	-1	-1	-1	3.8 ± 0.7	40.8 ± 5.0%	1.6 ± 0.3
2	+ 1	-1	-1	-1	4.8 ± 0.9	68.3 ± 1.8%	3.3 ± 0.4
3	-1	+ 1	-1	-1	5.7 ± 0.9	62.9 ± 1.3%	3.6 ± 0.5
4	+ 1	+ 1	-1	-1	3.6 ± 0.1	69.1 ± 2.9%	2.5 ± 0.1
5	-1	-1	+ 1	-1	3.8 ± 0.4	43.3 ± 5.5%	1.7 ± 0.3
6	+ 1	-1	+ 1	-1	3.3 ± 0.6	73.1 ± 2.4%	2.5 ± 0.3
7	-1	+ 1	+ 1	-1	5.7 ± 1.2	67.0 ± 1.4%	3.8 ± 0.4
8	+ 1	+ 1	+ 1	-1	3.4 ± 0.7	69.2 ± 2.7%	2.4 ± 0.3
9	-1	-1	-1	+ 1	5.2 ± 0.9	85.5 ± 1.0%	4.5 ± 0.4
10	+ 1	-1	-1	+ 1	3.2 ± 0.2	66.2 ± 2.7%	2.2 ± 0.2
11	-1	+ 1	-1	+ 1	5.4 ± 0.7	76.1 ± 2.1%	4.2 ± 0.4
12	+ 1	+ 1	-1	+ 1	3.7 ± 0.5	65.1 ± 2.0%	2.4 ± 0.3
13	-1	-1	+ 1	+ 1	5.3 ± 0.8	84.0 ± 2.2%	4.6 ± 0.5
14	+ 1	-1	+ 1	+ 1	3.6 ± 0.6	65.7 ± 2.1%	2.4 ± 0.3
15	-1	+ 1	+ 1	+ 1	4.0 ± 0.6	75.6 ± 1.7%	3.1 ± 0.3
16	+ 1	+ 1	+ 1	+ 1	3.3 ± 0.3	64.2 ± 2.0%	2.2 ± 0.2

^a^The initial cell seeding density was 5 × 10^5^ CD14^+^ cells in 1.5 ml differentiation medium containing 50 ng/ml GM-CSF and 50 ng/ml IL-4

^b^ + 1: the adding concentration of indicating cytokine was 50 ng/ml; − 1: no added cytokine

^c^Cell numbers in 1.5 ml differentiation medium and CD40^+^CD209^+^ percentage at day 5 (*n* = 3)

3 $$\textrm{CD}{40}^{+}\textrm{CD}{209}^{+}\textrm{DCs}/1.5\ \textrm{mL}\ \left(\times {10}^4\right)=2.94-{0.45}x_{1}+{0.09}x_{2}-{0.10}x_{3}+{0.26}x_{4}-{0.20}x_{1}x_{2}-{0.45}.x_{1}x_{4}-{0.05}x_{2}x_{3}-{0.31}x_{2}x_{4}-{0.03}x{3}x_{4}+{0.09}x_{1}x_{2}x_{3}+{0.43}x_{1}x_{2}x_{4}+{0.28}x_{1}x_{3}x_{4}-{0.15}x_{2}x_{3}x_{4}$$

In Eqs. 1, 2 and 3, *x*_*1*_ to *x*_*4*_ are coded variables for IL − 1β, IL-6, IL-7 and M-CSF, respectively, and only listed significant terms (*p* < 0.05). Equation 2 specifies that IL − 1β, IL-6, IL-7 and M-CSF had positive coefficients to enhance the percentage of CD40^+^CD209^+^ DCs in the total cells after differentiation. However, Eq. 1 indicated that IL − 1β and IL-7 had negative coefficients to decrease the total cell numbers after differentiation. Taken together, Eq. 3 indicated that the addition of IL-6 and M-CSF could increase CD40^+^CD209^+^ DC numbers after 5 days of differentiation in SF-DC Control medium.

### Concentration optimization of IL-6 and M-CSF-steepest ascent path

The steepest ascent path was determined by the coefficients in Eq. 3 to optimize the concentrations of IL − 1β and M-CSF for maximal CD40^+^CD209^+^ DCs differentiated from monocytes in SF-DC Control medium (Table 2). The total cell number, percentage of CD40^+^CD209^+^ DCs in the total cells and CD40^+^CD209^+^ DC number initially increased along the steepest ascent path, reaching 7.2 ± 1.0 × 10^4^ cells/1.5 mL, 74.5 ± 1.0% and 5.4 ± 0.7 × 10^4^ cells/1.5 mL at step 4 after a 5-day differentiation under the indicated condition, respectively. After step 4, there were no additional increases in total cell number, percentage of CD40^+^CD209^+^ DCs in the total cells or CD40^+^CD209^+^ DC number. Consequently, the optimal cytokine concentration for DC differentiation from monocytes was to add 1.4 ng/mL IL-6 and 3.0 ng/mL M-CSF to the SF-DC Control medium (named SF-DC Optimal medium).

**Table 2:** The concentrations of cytokines along the steepest ascent path and the numbers of CD40^+^CD209^+^ DCs differentiated from CD14^+^ monocytes^a^

Step	IL-6 (ng/ml)	M-CSF (ng/ml)	GM-CSF (ng/ml)	IL-4 (ng/ml)	Total cells (10^**4**^ cells)^**b**^	CD40^**+**^CD209^**+**^ (%) ^**b**^	CD40^**+**^CD209^**+**^ cells (10^**4**^ cells)^**b**^
1	0	0	50.0	50.0	4.1 ± 0.4	38.7 ± 6.1%	1.6 ± 0.1
2	0.5	1.0	50.0	50.0	6.8 ± 0.7	57.8 ± 1.2%	3.9 ± 0.6
3	0.9	2.0	50.0	50.0	6.9 ± 0.8	70.6 ± 2.0%	4.9 ± 0.8
**4**	**1.4**	**3.0**	**50.0**	**50.0**	**7.2 ± 1.0**	**74.5 ± 1.0%**	**5.4 ± 0.7**
5	1.9	4.0	50.0	50.0	7.1 ± 1.1	74.4 ± 2.9%	5.3 ± 0.6
6	2.3	5.0	50.0	50.0	6.8 ± 1.3	74.1 ± 0.5%	5.0 ± 0.9
7	2.8	6.0	50.0	50.0	7.0 ± 1.0	74.9 ± 2.1%	5.2 ± 0.9
8	3.3	7.0	50.0	50.0	6.9 ± 0.9	77.4 ± 2.2%	5.3 ± 0.8
9	3.7	8.0	50.0	50.0	6.8 ± 0.8	74.0 ± 2.7%	5.1 ± 0.9
10	4.2	9.0	50.0	50.0	6.3 ± 0.8	74.8 ± 2.2%	4.7 ± 0.8
11	4.7	10.0	50.0	50.0	6.4 ± 0.8	76.6 ± 2.8%	4.9 ± 0.7
12	5.9	12.5	50.0	50.0	6.6 ± 1.0	73.6 ± 2.2%	4.9 ± 0.7
13	7.0	15.0	50.0	50.0	6.6 ± 0.8	73.5 ± 2.0%	4.8 ± 0.8
14	9.4	20.0	50.0	50.0	6.7 ± 0.8	73.2 ± 2.8%	4.9 ± 1.0

^a^The initial cell seeding density was 5 × 10^5^ CD14^+^ cells in 1.5 ml differentiation medium

^b^Cell numbers in 1.5 ml differentiation medium and CD40^+^CD209^+^ percentage at day 5 (*n* = 3)

### Growth kinetics and morphology of DC differentiation from CD14^+^ monocytes

**Fig. 1:**
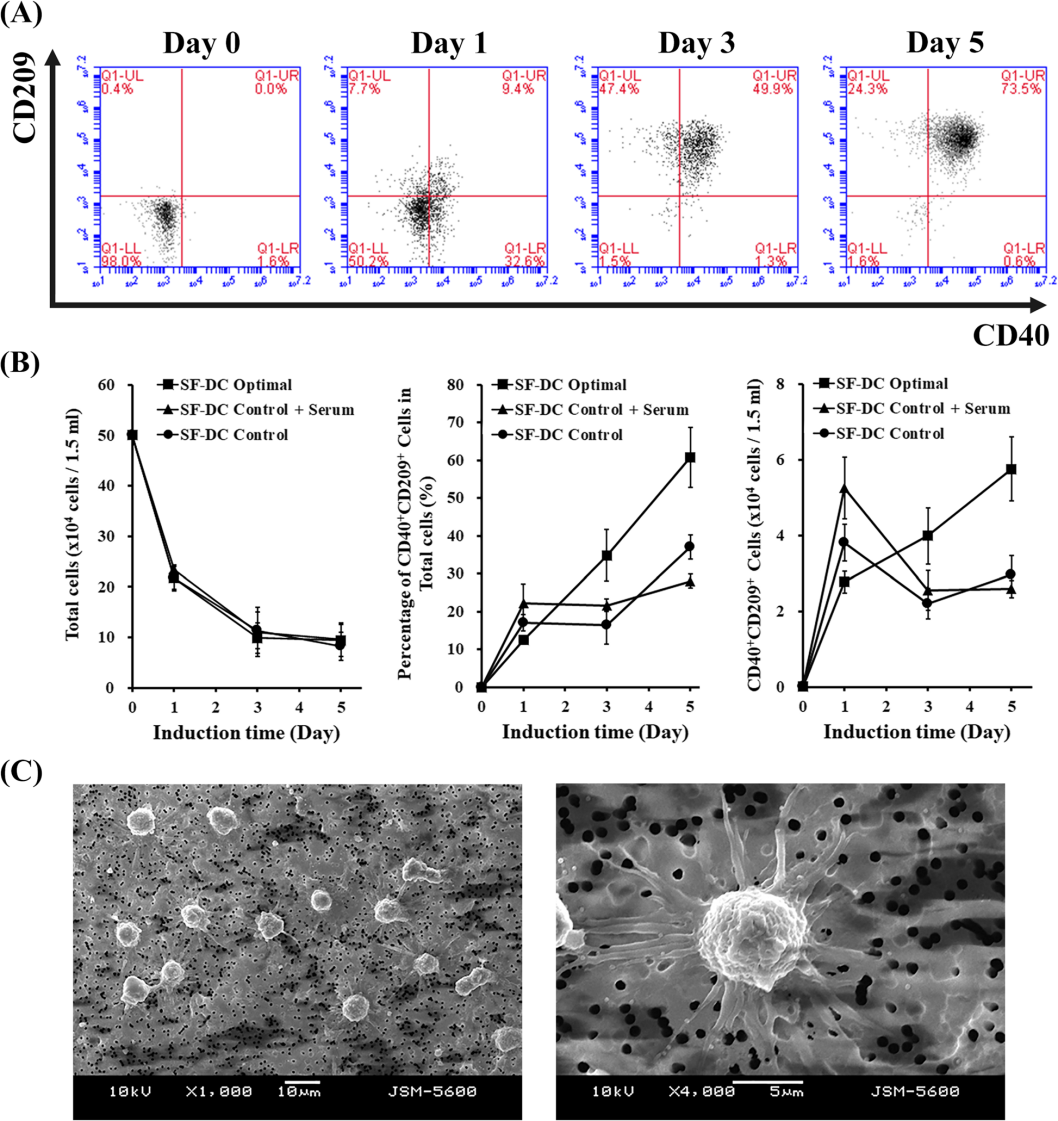
CD40 and CD209 profiles, growth kinetics and morphologies on DC differentiation from human monocytes. DCs were differentiated from CD14^+^ monocytes (5 × 10^5^ cells/1.5 mL) in SF-DC Optimal medium, SF-DC Control medium and SF-DC Control + Serum medium for 5 days. **A** The differentiated cells in SF-DC Optimal medium at the indicated time points were stained with CD40 and CD209-PE. The numbers within the dot plots represent the percentages of the indicated cells in the total cell population by flow cytometry analysis. **B** The accumulated total cell number, percentage of CD40^+^CD209^+^ DCs in the total cells and CD40^+^CD209^+^ DC number differentiated from CD14^+^ monocytes at the indicated time points were determined by flow cytometry analysis (*n* = 5). (I) Representative morphologies of the differentiated DCs on Day 5 in SF-DC Optimal medium under scanning electron microscopy observation. Scale bars are 10 μm (left figure) and 5 μm (right figure)

After developing the SF-DC Optimal medium for DC differentiation from human CD14^+^ monocytes, the total cell numbers, the percentage of CD40^+^CD209^+^ DCs in the total cells and differentiated CD40^+^CD209^+^ DC numbers differentiated in the SF-DC Optimal medium were monitored and compared with those in the SF-DC Control medium and SF-DC Control + Serum medium (Fig. 1).

Our results showed that freshly isolated CD14^+^ monocytes did not express CD40 or CD209 (Fig. 1A, Day 0). As the differentiation time increased, the total cell numbers in the SF-DC Optimal medium, SF-DC Control medium and SF-DC Control + Serum medium all decreased rapidly and continuously (Fig. 1B). This phenomenon means that the DC differentiation process inevitably accompanies apoptosis. Meanwhile, the percentage of CD40^+^CD209^+^ DCs in the total cells and differentiated CD40^+^CD209^+^ DC numbers began to increase (Fig. 1A). After 5 days of differentiation, the percentages of CD40^+^CD209^+^ DCs in the total cultured cells and the CD40^+^CD209^+^ DC density in the SF-DC Optimal medium on Day 5 were 65.2 ± 7.9% and 5.75 ± 0.84 × 10^4^ cells/1.5 mL, respectively, and reached approximately twofold greater than those in the SF-DC Control medium and SF-DC Control + Serum medium (Fig. 1B). Importantly, the differentiated CD40^+^CD209^+^ DCs in the SF-DC Optimal medium exhibited dendritic-like tentacle morphology (Fig. 1C). This finding indicated that IL-6 and M-CSF were necessary for DC differentiation from monocytes and exhibited a synergistic effect with GM-CSF and IL-4 to enhance DC differentiation with correct morphology under serum-free condition.

### Expression of DC-related surface markers and stimulation by TNF-α or LPS

**Fig. 2:**
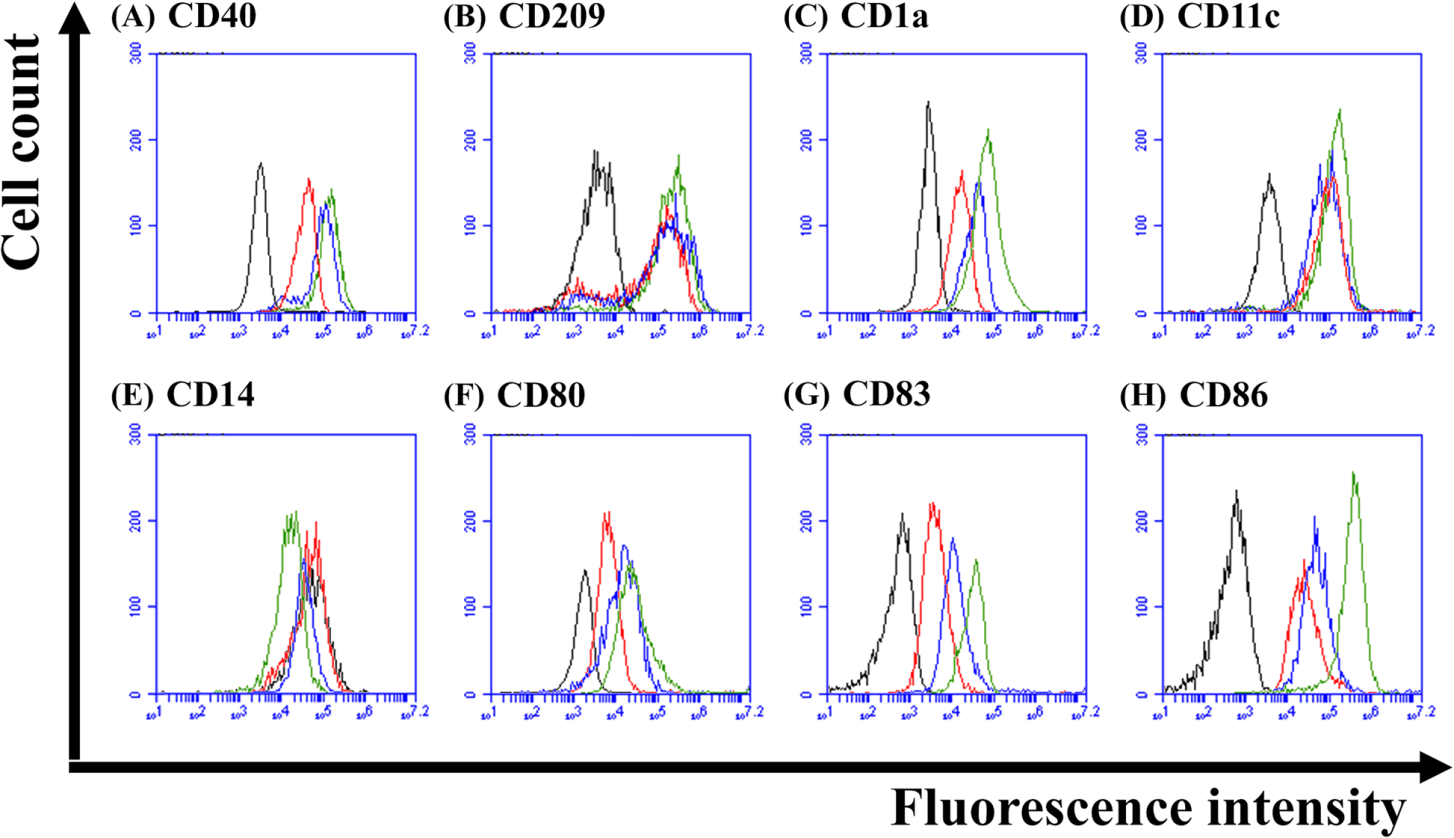
Representative histograms of DC-related surface marker expressions on differentiated DCs from human monocytes in SF-DC Optimal medium. DCs were differentiated from CD14^+^ monocytes (5 × 10^5^ cells/1.5 mL) in SF-DC Optimal medium for 5 days (Day 0 to Day 5), and then, 20 ng/ml TNF-α or 1 μg/mL lipopolysaccharide (LPS) was added to stimulate maturation for 2 days (Day 5 to Day 7). After differentiation, the generated cells at the indicated time points (Day 0: black peaks; Day 5: red peaks; Day 7 with TNF-α: blue peaks; Day 7 with LPS: green peaks) were stained with antibodies against (**A**) CD40, **B** CD209, **C** CD1a, **D** CD11c, **E** CD14, **F** CD80, **G** CD83 and **H** CD86. The histogram of the indicated surface marker expression in the total cell population was analyzed by flow cytometry

**Fig. 3:**
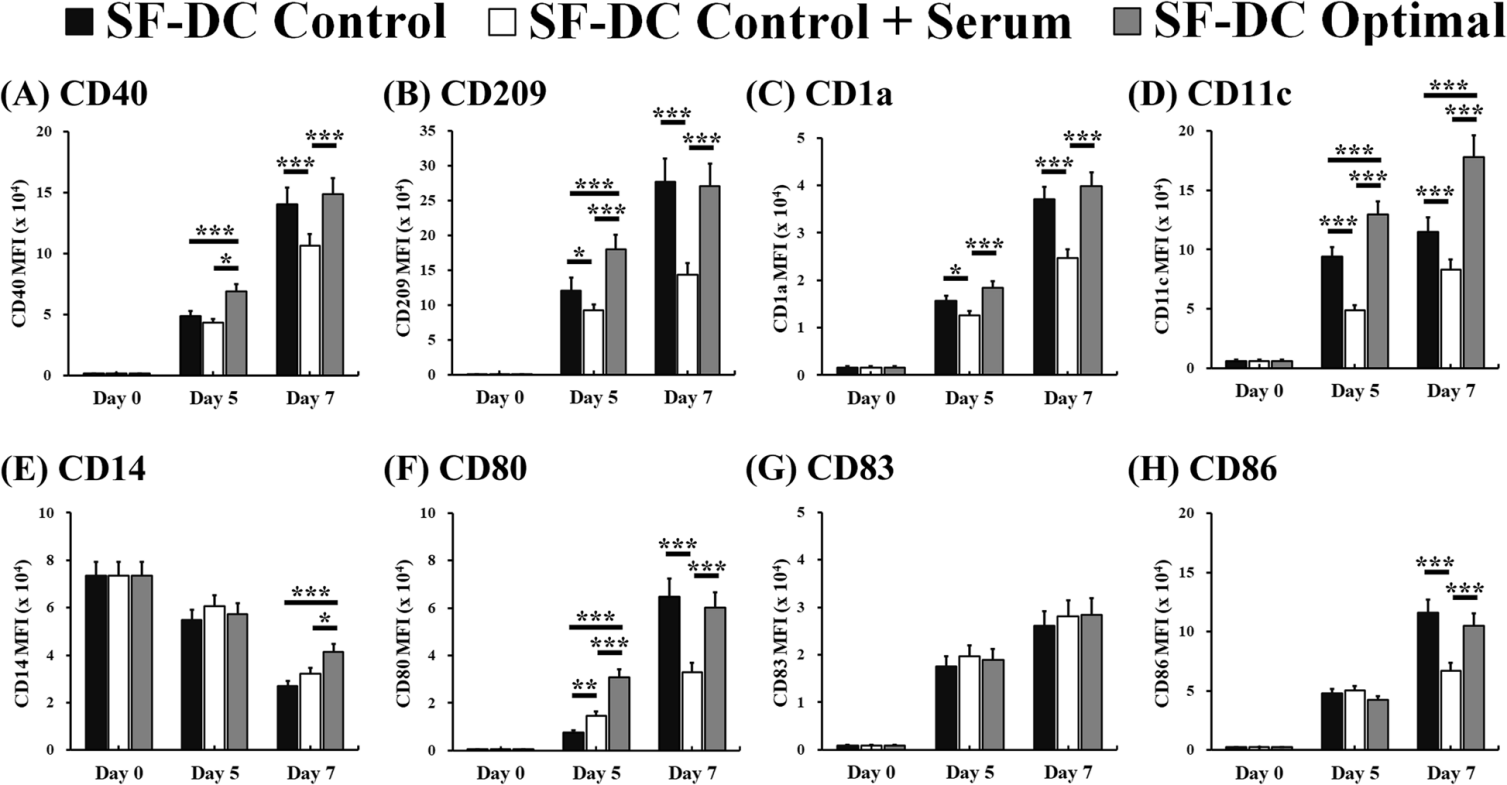
Expression of DC-related surface markers on differentiated DCs from human monocytes. DCs were differentiated from CD14^+^ monocytes (5 × 10^5^ cells/1.5 mL) in SF-DC Optimal medium, SF-DC Control medium and SF-DC Control + Serum medium for 5 days (Day 0 to Day 5), and then, 20 ng/ml TNF-α was added to the corresponding medium to stimulate maturation for 2 days (Day 5 to Day 7). After differentiation, the generated cells at the indicated time points were stained with antibodies against (**A**) CD40, **B** CD209, **C** CD1a, **D** CD11c, **E** CD14, **F** CD80, **G** CD83 and **H** CD86. The mean fluorescence intensity (MFI) of the indicated cells in the total cell population was analyzed by flow cytometry. *, ** and *** represent significant differences of *p* < 0.05, *p* < 0.01 and *p* < 0.005, respectively (*n* = 5)

**Fig. 4:**
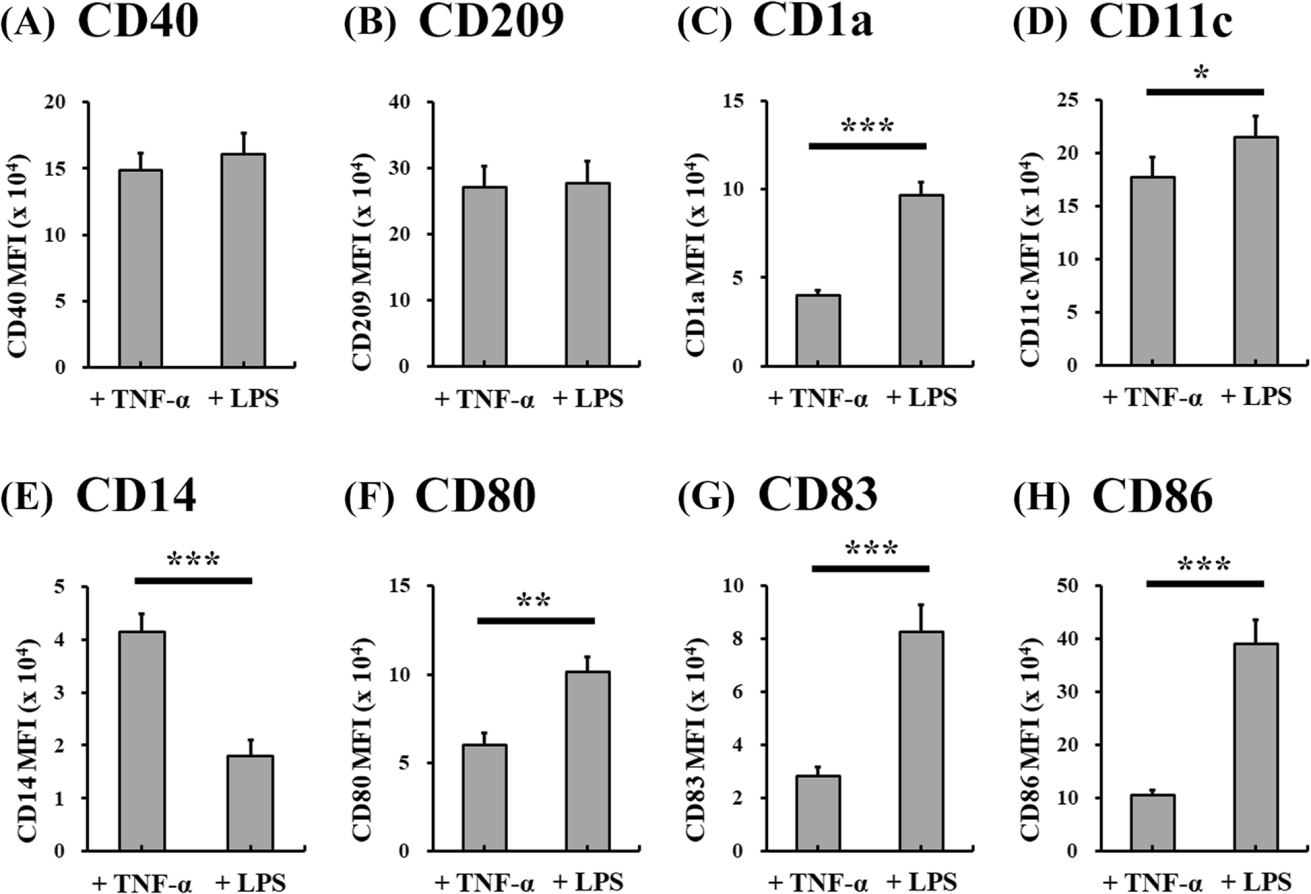
Comparison of DC-related surface marker expression on differentiated DCs from human monocytes in SF-DC Optimal medium with TNF-α and lipopolysaccharide stimulation. DCs were differentiated from CD14^+^ monocytes (5 × 10^5^ cells/1.5 mL) in SF-DC Optimal medium for 5 days (Day 0 to Day 5), and then, 20 ng/ml TNF-α or 1 μg/mL lipopolysaccharide (LPS) was added to stimulate maturation for 2 days (Day 5 to Day 7). After differentiation, the generated cells on Day 7 were stained with antibodies against (**A**) CD40, **B** CD209, **C** CD1a, **D** CD11c, **E** CD14, **F** CD80, **G** CD83 and (**H**) CD86. The mean fluorescence intensity (MFI) of the indicated cells in the total cell population was analyzed by flow cytometry. *, ** and *** represent significant differences of *p* < 0.05, *p* < 0.01 and *p* < 0.005, respectively (*n* = 5)

After confirming the importance and necessity of IL-6 and M-CSF for DC differentiation, the other DC-related surface marker expression levels, including CD1a, CD11c, CD14, CD40, CD80, CD83, CD86 and CD209, of differentiated DCs before (on Day 5) and after (on Day 7) stimulation by TNF-α or LPS in the SF-DC Optimal medium were also checked and compared with those in the SF-DC Control medium and SF-DC Control + Serum medium (Figs. 2, 3 and 4).

After 5 days of differentiation in the SF-DC Optimal medium, the expression of the abovementioned DC-related surface markers, except CD14, was obviously expressed on the differentiated cells compared to those on the initial CD14^+^ monocytes (on Day 0). In addition, the expression of these DC-related surface markers on the differentiated cells in the SF-DC Optimal medium was higher or comparable to that in the SF-DC Control medium and SF-DC Control + Serum medium. Differentiation of monocytes into DCs was accompanied by downregulation of CD14 expression. These decreasing trends were observed in all differentiation methods (Fig. 3E). After stimulation with TNF-α or LPS, our results showed that the expression of CD80, CD83 and CD86 on the differentiated DCs (on Day 7) was significantly higher than that on DCs before stimulation (on Day 5) (Figs. 2 and 3). It is worth noting that the differentiated DCs (on Day 7) stimulated by LPS exhibited more obvious DC-related surface marker expressions (such as CD1a, CD80, CD83 and CD86) and lower CD14 expression than those by TNF-α (Figs. 2 and 4). These results demonstrated that the differentiated DCs expressed correct surface markers and could be further activated to mature DCs by TNF-α and LPS, and the differentiated DCs in the SF-DC Optimal medium with LPS stimulation are competent to fully mature into DCs.

### Endocytosis analysis

**Fig. 5:**
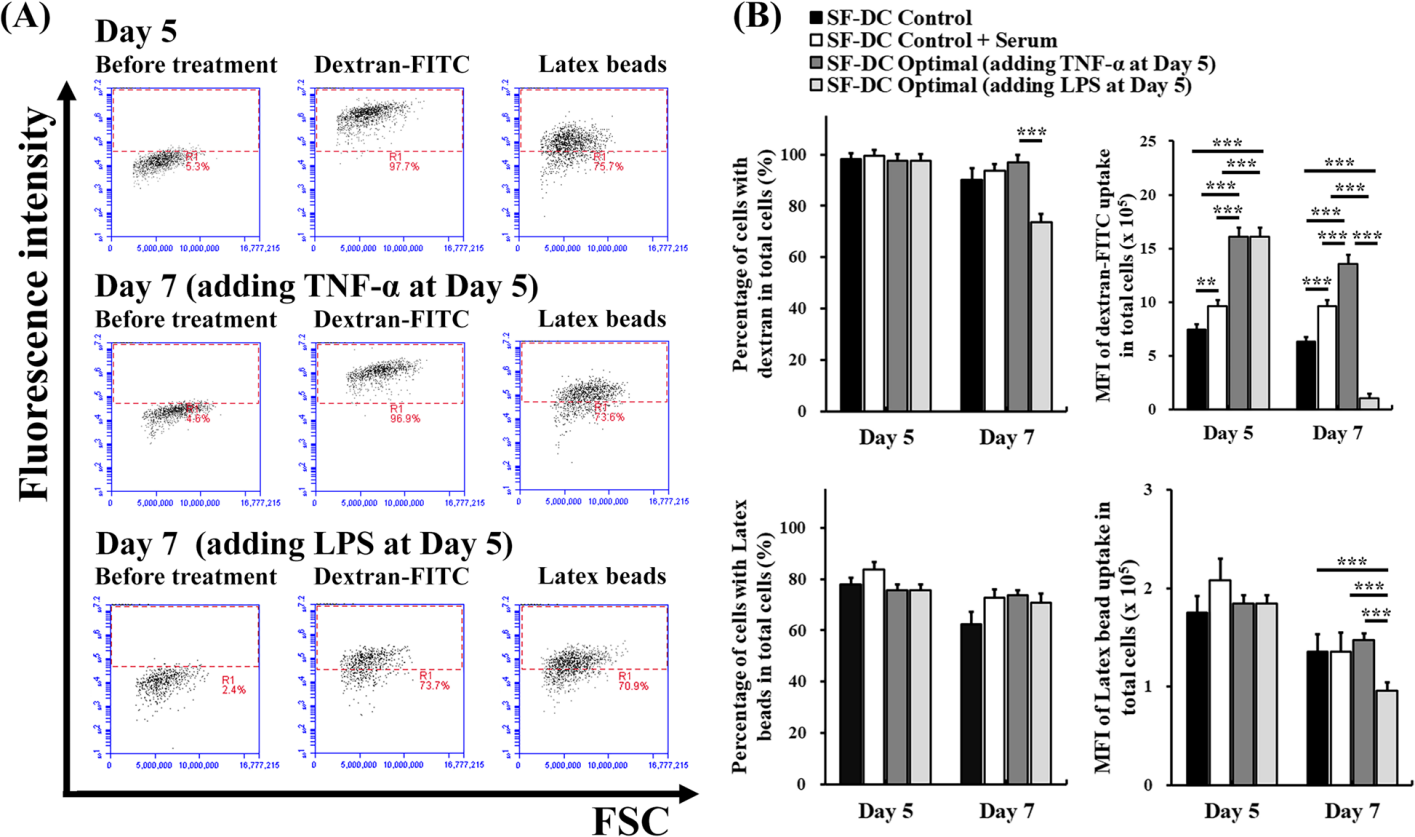
Endocytosis analysis of the differentiated DCs from monocytes. DCs were differentiated from CD14^+^ monocytes (5 × 10^5^ cells/1.5 mL) in SF-DC Optimal medium, SF-DC Control medium and SF-DC Control + Serum medium for 5 days (Day 0), and then, 20 ng/ml TNF-α or 1 μg/mL lipopolysaccharide (LPS) was added to the corresponding medium to stimulate maturation for 2 days (Day 5 to Day 7). After differentiation, the cells were treated with 1 mg/mL dextran-fluorescein isothiocyanate (dextran-FITC) or 1 mg/mL fluorescent latex beads and stained with a CD209 fluorescence antibody. The endocytosis ability was determined by the CD209^+^ cells that expressed the fluorescence of dextran-FITC or latex beads using flow cytometry. **A** Representative endocytosis analysis of the differentiated DCs in SF-DC Optimal medium before (Day 5) and after (Day 7) TNF-α or LPS stimulation for maturation by flow cytometry. **B** The percentages and the mean fluorescence intensity (MFI) of the cells with dextran-FITC or fluorescent latex bead uptake in total CD209^+^ DCs at the indicated time points were analyzed by flow cytometry. ** and *** represent significant differences of *p* < 0.01 and *p* < 0.005, respectively (*n* = 3)

The endocytosis abilities of differentiated DCs were analyzed by treatment with dextran and latex beads to represent the uptake of small and large molecules (particles), respectively (Fig. 5A). The percentage results showed that almost all differentiated DCs and approximately 75% of differentiated DCs exhibited endocytotic uptake of dextran and latex beads, respectively, in the SF-DC Optimal medium (on Day 5) and were similar to those in the SF-DC Control medium and SF-DC Control + Serum medium. Importantly, the mean fluorescence intensity (MFI) results indicated that the uptake amount of dextran per differentiated DC on Day 5 in the SF-DC Optimal medium (MFI: 16.1 × 10^5^) was significantly higher than that in the SF-DC Control medium (MFI: 7.5 × 10^5^) and SF-DC Control + Serum medium (MFI: 9.6 × 10^5^) (Fig. 5B). In addition, the uptake MFIs of the differentiated DCs on Day 7 were decreased compared with those on Day 5 due to maturation by TNF-α or LPS stimulation. It is worth noting that the uptake MFIs of the differentiated DCs on Day 7 stimulated by LPS (MFI: 1.0 × 10^5^ and 0.9 × 10^5^ for uptake of dextran and latex beads, respectively) were significantly lower than those by TNF-α (MFI: 13.5 × 10^5^ and 1.4 × 10^5^ for uptake of dextran and latex beads, respectively) (Fig. 5B). This meant that the DCs differentiated in the SF-DC Optimal medium maintained excellent endocytotic ability and maintained the ability to be activated to mature, especially by stimulation of LPS.

### Mixed Leukocyte Reaction (MLR)

**Fig. 6:**
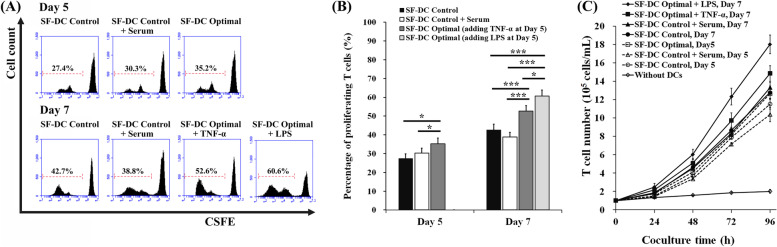
Mixed leukocyte reaction of the differentiated DCs from monocytes. DCs were differentiated from CD14^+^ monocytes (5 × 10^5^ cells/1.5 mL) in SF-DC Optimal medium, SF-DC Control medium and SF-DC Control + Serum medium for 5 days (Day 0 to Day 5), and then, 20 ng/ml TNF-α or 1 μg/mL lipopolysaccharide (LPS) was added to the corresponding medium to stimulate maturation for 2 days (Day 5 to Day 7). After differentiation, CD209^+^ DCs were isolated from the differentiated cells using anti-CD209 microbeads on a VarioMACS separator. Then, the isolated CD209^+^ DCs were cocultured with allogenic CFSE-stained CD3^+^ T cells at a ratio of 1:2 for mixed leukocyte reactions. After 4 days of coculture, total cells were harvested, and proliferating CD3^+^ T cells were determined using flow cytometry. **A** Representative analysis of CFSE expression in cocultured cells. The cells in the dotted boundary represent proliferating CD3^+^ T cells with decayed CFSE expression. **B** The percentages of proliferating CD3^+^ T cells among the total CD3^+^ T cells. * and *** represent a significant difference of *p* < 0.05 and *p* < 0.005, respectively (*n* = 3). **C** Growth kinetics of CD3^+^ T-cell expansion stimulated by coculture with various differentiated DCs (*n* = 3). The initial CD3^+^ T-cell density was 1 × 10^5^ cells/mL

The immunostimulatory capacity of DCs is an important and practical indicator in clinical use. In this study, the T cell expansion stimulated by DCs was evaluated by an MLR assay using carboxyfluorescein succinimidyl ester (CFSE) staining (Fig. 6A and B) and T cell number counting (Fig. 6C). Before (on Day 5) and after stimulation (on Day 7), the coculture results showed that the differentiated DCs in the SF-DC Optimal medium (percentage of proliferating T cells on Day 5: 35.2%, and Day 7: 52.6 and 60.6% by stimulation of TNF-α and LPS, respectively) exhibited superior capacities to trigger CD3^+^ T cell proliferation than those in the SF-DC Control medium (percentage of proliferating T cells on Day 5: 27.4% and Day 7: 42.7% by stimulation of TNF-α) and DF-DC Control + Serum medium (percentage of proliferating T cells on Day 5: 30.3% and Day 7: 38.8% by stimulation of TNF-α). In addition, the results indicated that the immunostimulatory capacity of the differentiated DCs could be further enhanced after TNF-α and LPS stimulation. After 96 h of coculture, the expansion fold of CD3^+^ T cell number cocultured with the differentiated DCs in the SF-DC Optimal medium on Day 5, Day 7 stimulation by TNF-α and Day 7 stimulation by LPS was approximately 12.6-, 14.9- and 18.0-fold, respectively (Fig. 6C). This result once again demonstrated that the differentiated DCs in the SF-DC Optimal medium maintained the ability to mature by TNF-α or LPS stimulation and exhibited a strong immunostimulatory capacity.

### Cytokine secretion

**Fig. 7:**
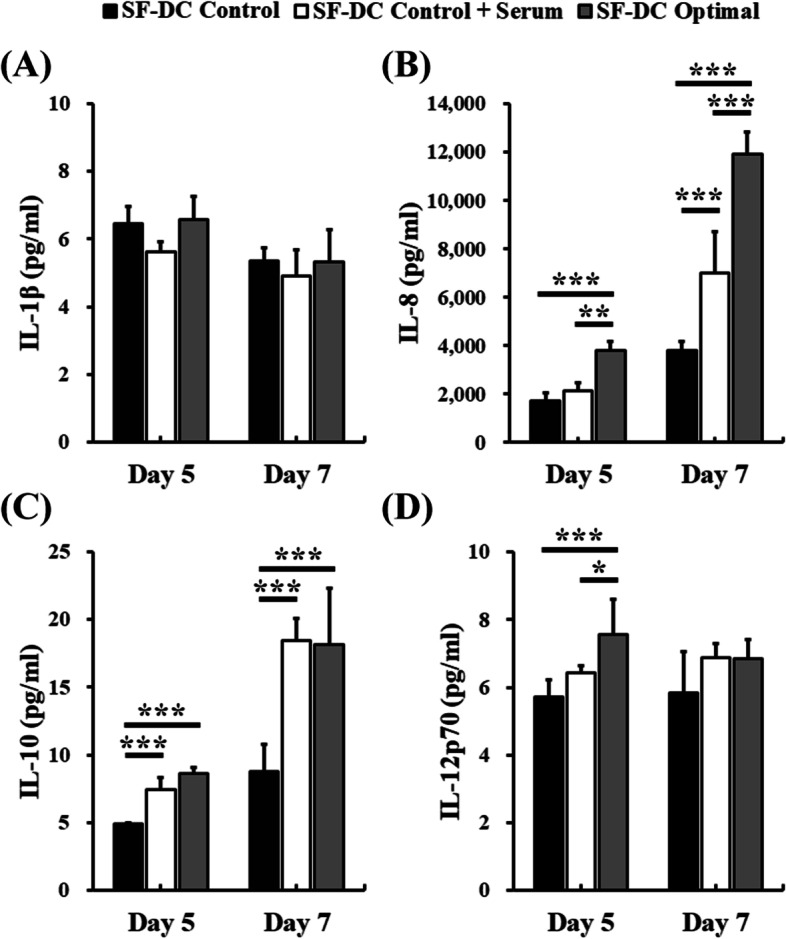
Cytokine secretion of the differentiated DCs from monocytes. DCs were differentiated from CD14^+^ monocytes (5 × 10^5^ cells/1.5 mL) in SF-DC Optimal medium, SF-DC Control medium and SF-DC Control + Serum medium for 5 days (Day 0 to Day 5), and then, 20 ng/ml TNF-α was added to the corresponding medium to stimulate maturation for 2 days (Day 5 to Day 7). After differentiation, the conditioned media at the indicated time points were collected, and the secreted amounts of (**A**) IL-1β, **B** IL-8, **C** IL-10 and **D** IL-12p70 were analyzed by flow cytometry. *, ** and *** represent significant differences of *p* < 0.05, *p* < 0.01 and *p* < 0.005, respectively (*n* = 3)

**Fig. 8:**
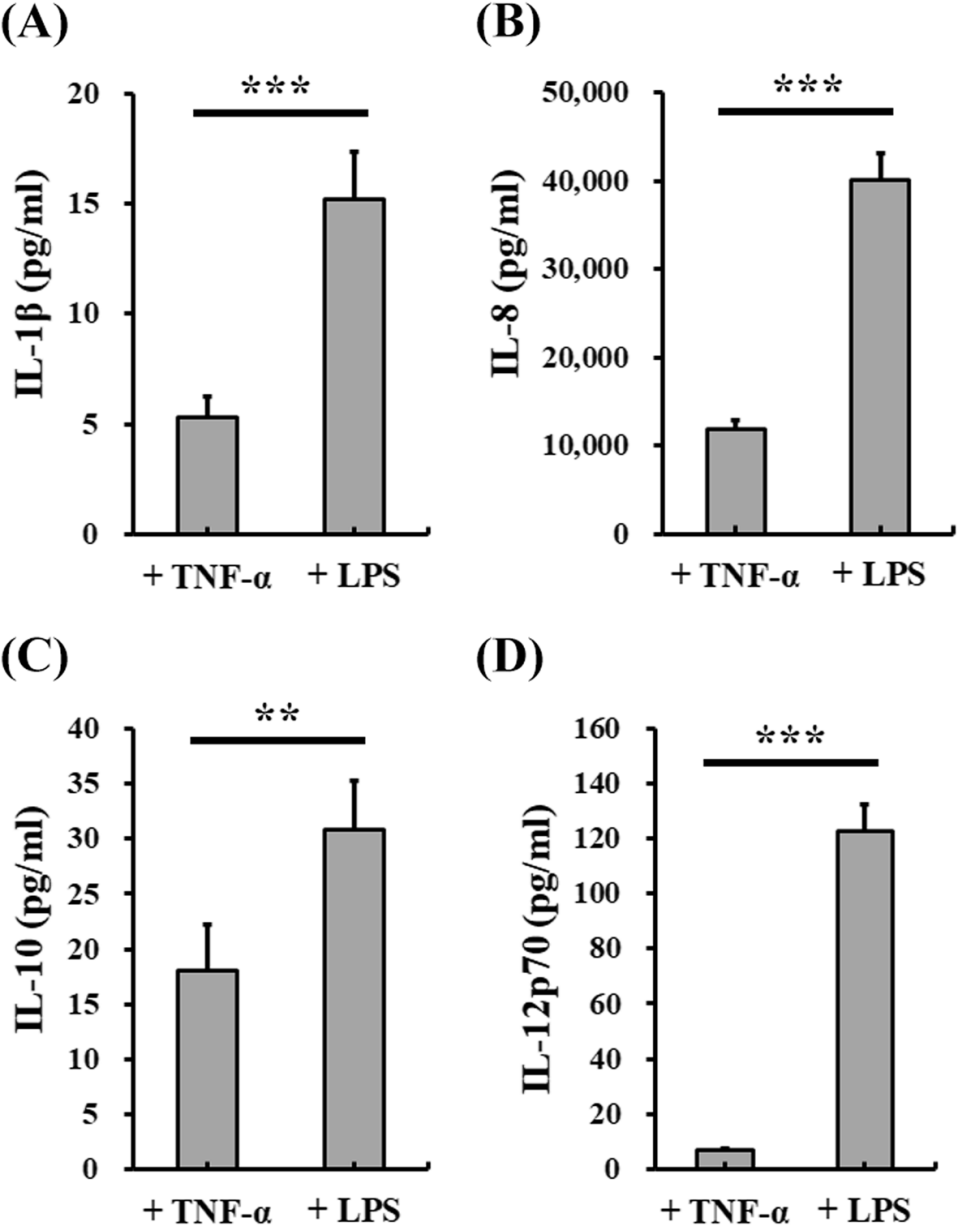
Comparison of cytokine secretion of the differentiated DCs from human monocytes in SF-DC Optimal medium with TNF-α and lipopolysaccharide stimulation. DCs were differentiated from CD14^+^ monocytes (5 × 10^5^ cells/1.5 mL) in SF-DC Optimal medium for 5 days (Day 0 to Day 5), and then, 20 ng/ml TNF-α or 1 μg/mL lipopolysaccharide (LPS) was added to stimulate maturation for 2 days (Day 5 to Day 7). After differentiation, the conditioned media on Day 7 were collected, and the secreted amounts of (**A**) IL-1β, **B** IL-8, **C** IL-10 and **D** IL-12p70 were analyzed by flow cytometry. ** and *** represent significant differences of *p* < 0.01 and *p* < 0.005, respectively (*n* = 3)

Cytokines play an important and complex role in the regulation of the immune system. In this study, the cytokines secreted from the differentiated DCs in the various DC differentiation media before and after stimulation were determined by cytometric bead array (Figs. 7 and 8). Our results showed that IL − 1β, IL-8, IL − 10 and IL − 12p70 secreted from differentiated DCs on Day 5 were obviously detected in the conditioned SF-DC Optimal medium, SF-DC Control medium and SF-DC Control + Serum medium. Importantly, the differentiated DCs in the SF-DC Optimal medium on Day 5 secreted higher amounts of IL-8, IL − 10 and IL − 12p70 than those in the SF-DC Control or SF-DC Control + medium (Fig. 7). In addition, after TNF-α stimulation, the secretion amount of IL-8 and IL − 10 from differentiated DCs on Day 7 in the SF-DC Optimal medium significantly increased and was significantly higher than those in the SF-DC Control or SF-DC Control + medium. The secretion of IL − 1β in all media was maintained at a similar level. The secretion amounts of IL-8 and IL − 10 by the differentiated DCs in the SF-DC Control medium were significantly lower than those in the SF-DC Control + Serum medium. These results indicated that the components in the SF-DC Control medium could not completely replace the role of serum for DC differentiation, and the addition of IL-6 and M-CSF was necessary to differentiate functional DCs under serum-free condition. It is worth noting that the differentiated DCs (on Day 7) stimulated by LPS secreted significantly higher amount of IL − 1β, IL-8, IL − 10 and IL − 12p70 than those by TNF-α (approximate 2.6-, 3.4-, 1.7- and 17.9-fold) (Fig. 8). These results demonstrated that the differentiated DCs secreted DC-related cytokines and the differentiated DCs in the SF-DC Optimal medium could be furtherly stimulated by LPS to mature.

Combined with the growth kinetics, surface marker expression, endocytosis analysis, T-cell immunostimulatory capacity and cytokine secretion, these results indicated that the SF-DC Optimal medium containing IL-6 and M-CSF developed in this study could be efficiently used to differentiate monocytes into DCs with correct function.

## Discussion

DCs are one of the major APCs in the human immune system and have the strongest abilities to perform phagocytosis, present antigens and stimulate T proliferation. Therefore, DCs are considered to play an important role in immunotherapy. However, insufficient cell number and low function of DCs are the major bottleneck in bench research and immunotherapy application. Currently, the common method to differentiate DCs from monocytes is to add high concentrations (80–100 ng/mL) of GM-CSF and IL-4 under serum-containing culture condition. However, the number and the function of the differentiated DCs still need to be improved. Therefore, the development of a cytokine-optimized and serum-free DC differentiation medium that can efficiently differentiate DCs from monocytes is important and urgent for DC research and application. In our previous study, a SF-DC Control medium was proposed [21]. SF-DC Control medium contained high concentrations of GM-CSF (80 ng/ml) and IL-4 (80 ng/ml), and thirteen serum substitutes were used to replace the role of serum. In the present study, our results indicated that although the growth kinetics of differentiated DCs in the SF-DC Control medium were very similar to those in the SF-DC Control + Serum medium, some functions, such as small molecule uptake and cytokine secretion, were different. These results indicated that the formula of the cytokine cocktail and serum substitutes in the SF-DC Control could not completely replace serum. One cytokine might act on different types of cells and have different effects, depending on the target cells, its concentration, and the presence of other cytokines. Serum is a mixture containing many trace cytokines (such as IL-6 and M-CSF) that may benefit or inhibit DC differentiation. To further increase the cell number and improve the function of differentiated DCs under serum-free condition, fifteen cytokines were screened and added to SF-DC Control medium using systematic and statistical methods to test their effect on DC differentiation in this study [22, 23, 33–39]. Finally, a SF-DC Optimal medium was proposed, and the number and the functions of differentiated DCs in the SF-DC Optimal medium were analyzed to compare with those in the SF-DC Control medium and SF-DC Control + Serum medium.

SF-DC Optimal medium comprised of RPMI 1640 medium, the same thirteen serum substitutes as SF-DC Control medium, lower concentrations of GM-CSF (50 ng/ml) and IL-4 (50 ng/ml), and extra trace amounts of IL-6 (1.4 ng/ml) and M-CSF (3 ng/ml). Previous literatures demonstrated that M-CSF can enhance MHC-restricted antigen presentation in DCs and IL-6 had the anti-apoptotic effect and were served as a common maturation cytokine cocktail supplement [39, 40]. Our results demonstrated that the cytokine cocktail was modified successfully, which generated more DCs with superior or comparable functions compared to the serum-containing method. According to other reports, the concentrations of M-CSF and IL-6 in serum have been analyzed [41–44] and these cytokines might be beneficial for cell development even though the concentrations were very low, especially in serum-free culture condition. Hence, it is necessary to modify the medium composition by the addition of trace cytokines. This research is the first report finding that a combination of an extra low concentration of IL-6 and M-CSF exhibited a synergistic and enhancive effect with GM-CSF and IL-4 to efficiently differentiate more human DCs from monocytes and enhance functions of DCs, such as phagocytosis ability, cytokine secretion and mixed lymphocyte reaction under serum-free condition.

After 5-day differentiation in SF-DC Optimal medium, the average number of differentiated DCs was 5.75 ± 0.84 × 10^4^ cells from the initial 5.00 × 10^5^ monocytes, which was two times more than those in SF-DC Control medium and SF-DC Control + Serum medium. The differentiated DCs highly expressed DC-related surface markers. In addition, the expression of DC-related surface markers was significantly upregulated after TNF-α or LPS stimulation. This means that the differentiated DCs maintained the ability to mature. Notably, most DC-related surface marker expression of DCs after LPS stimulation in the SF-DC Optimal medium was significantly higher than that after TNF-α stimulation. Taking the growth kinetics and surface marker expression together, these results demonstrated that trace amounts of IL-6 and M-CSF were necessary for DC differentiation from monocytes under serum-free condition and could induce DCs to be more mature than those without IL-6 and M-CSF. Generally, CD14 expression should be downregulated abruptly during the DC differentiation process. However, our results showed that even though all DC-related surface markers were obviously present, there was only a slight decrease in CD14 expression (on Day 5 or on Day 7 stimulation by TNF-α) compared to monocytes (on Day 0). Several reports have indicated that monocyte-derived DCs may retain CD14 expression in an intermediate grade or are present at variable levels according to the environment or steady state in tissue [24, 25]. The retention of CD14 expression in the differentiated DCs in the SF-DC Optimal medium might be caused by the addition of IL-6 [26]. The other reason might be that TNF-α could suppress some functions of DCs, especially in the IL-4/GM-CSF/TNF-α protocol and could not induce full maturation of DCs [27]. In addition, previous studies had proven that CD14 expression and binding affinity of LPS with toll-like receptor 4 (TLR4) are highly correlated [28]. Therefore, the presence of CD14 indicated that differentiated DCs in SF-DC Optimal medium might exhibit high affinity for LPS for maturation.

For functional analysis of DCs, the differentiated DCs in SF-DC Optimal medium exhibited stronger phagocytosis ability and immunostimulatory capacity than those in SF-DC Control and SF-DC Control + Serum media. These results indicated that IL-6 and M-CSF exhibited a synergistic effect with GM-CSF and IL-4 to enhance the abilities of DCs to stimulate T-cell proliferation and proved that the differentiated DCs in SF-DC Optimal medium were more mature than those in SF-DC Control and SF-DC Control + Serum media.

For cytokine secretion analysis, the differentiated DCs in SF-DC Optimal medium on Day 7 stimulated by TNF-α showed an equivalent ability for IL − 12p70 and IL − 1β secretion but a higher ability for IL-8 and IL − 10 secretion compared to those in the SF-DC Control medium. In addition, the cytokine secretion abilities, except IL-8, of the differentiated DCs in the SF-DC Optimal medium stimulated by TNF-α were much more similar to those in the SF-DC Control + Serum medium. Importantly, the differentiated DCs in the SF-DC Optimal medium on Day 7 stimulated by LPS released significantly higher amount of IL − 12p70 and higher ratio of IL − 12p70/IL − 10 than by TNF-α. The polarization of the immune response toward a type 1 T helper (Th1) cell or a Th2 cell profile is mediated by the cytokines secreted from DCs following antigen presentation and interaction with T cells. Our results indicated that the differentiated DCs in the SF-DC Optimal medium tend to be immunogenic DCs (might be efficient in inducing Th1 polarization) by stimulation of LPS (higher ratio of IL − 12p70/IL-10) and tend to be tolerogenic DCs (might be efficient in inducing Th2 polarization) by stimulation of TNF-α (lower ratio of IL-12p70/IL-10) [29–31]. Immunogenic DCs and tolerogenic DCs both play important roles in basic research and immunotherapy. Several reports have shown that the microenvironment of DCs modulates subtype differentiation and CD1a expression. The two subsets of CD1a^+^ (designated mDC1) and CD1a^−^ (designated mDC2) monocyte-derived DCs have been described to have different profiles of cytokine secretion that result from the existence of serum lipoproteins [26, 32]. CD1a^+^ and CD1a^−^ DCs stand out for their capability to secrete high amounts of IL-12p70 and IL-10, respectively [32]. Therefore, the differentiated DCs in SF-DC Optimal medium stimulated by TNF-α tend to secrete IL-10 with low expression on CD1a, which might correspond to the features of mDC2s. On the other hand, the differentiated DCs in SF-DC Optimal medium stimulated by LPS tend to secrete IL-12p70 with high expression on CD1a, which might correspond to the features of mDC1s.

The differentiated DCs in SF-DC Optimal medium secreted high amounts of IL-8, which was caused by the addition of IL-6 and M-CSF, might have different functions compared to other DCs. IL-8 plays a complex role in the immune system. IL-8 is a chemokine that is initially characterized by leukocyte chemotaxis and is well known to possess proangiogenic and tumorigenic properties [45, 46]. Macrophages, epithelial cells, endothelial cells and tumor cells are common IL-8 producers, and DCs have been observed to generate IL-8 in an autocrine or paracrine fashion in the intratumoral setting [47]. In addition to IL-8 generation, DCs also express receptors, such as CXCR1 and CXCR2, which can be downregulated by the IL-8 microenvironment caused by autocrine signaling [48]. According to the effect of IL-8, therefore, the IL-8 produced from tumor cells not only affects metastasis and angiogenesis [49] but also attracts and retains DCs to repress their function by tumor-derived factors, which do not functionally affect T cell stimulation activity but restrict the migration ability of antigen-presenting cells [50]. Although IL-8 produced by tumor cells may affect the antigen-presenting ability of DCs, IL-8 also plays an important role in the immune response by its leukocyte chemotaxis. IL-8 secreted from DCs itself can regulate its receptor presentation and attract neutrophils, which is dependent on IL-8 levels [47, 50].

## Conclusions

Taken together, our results are the first to demonstrate that IL-6 and M-CSF exhibit synergistic effects with GM-CSF and IL-4 on DC differentiation and that SF-DC Optimal differentiation medium can efficiently generate a large number of functional DCs for basic research and clinical application.

## Methods

### Purification of CD14^+^ monocytes

Cord blood (CB) samples were collected and processed according to governmental regulations -“Guidelines for collection and use of human specimens for research”, Ministry of Health and Welfare, Taiwan and after obtaining approval from the institutional review board of Taoyuan General Hospital, Ministry of Health and Welfare, Taiwan. Briefly, after obtaining the mother’s consent, CB was harvested and processed within 24 h. Mononuclear cells (MNCs) were isolated by Ficoll-Paque (Amersham Biosciences, Uppsala, Sweden) density gradient centrifugation. Fresh CD14^+^ monocytes were purified with CD14 microbeads by a Mitenyi VarioMACS device (Miltenyi Biotec Gmbh, Bergisch Gladbach, Germany) according to the manufacturer’s instructions.

### Formulations of DC differentiation media

In this study, three formulations of media, SF-DC Control, SF-DC Control + Serum and SF-DC Optimal, were used, and their performances in inducing DCs from CD14^+^ monocytes were compared. SF-DC Control medium was developed and described previously as RPMI 1640 medium (Gibco, Carlsbad, CA) supplemented with thirteen serum substitutes (including 1.5 mg/ml human serum albumin (CSL Behring, King of Prussia, PA), 4.39 μg/ml human insulin (Sigma–Aldrich, St Louis, MO), 60 μg/ml human transferrin (Sigma–Aldrich), 25.94 μM 2-mercaptoethanol (Sigma–Aldrich), 1 mg/ml fetuin (Sigma–Aldrich), 0.013 μg/ml biotin (Sigma–Aldrich), 0.108 mM sodium pyruvate (Sigma–Aldrich), 0.136 mM L-glutamine (Sigma–Aldrich), 2.06 U/ml heparin (Sigma–Aldrich), 0.072 mM ascorbic acid (Sigma–Aldrich), 1% v/v nonessential amino acids (Sigma–Aldrich), 10 mM β-glycerolphosphate (Sigma–Aldrich), 10 nM dexamethasone (Sigma–Aldrich)), cytokine cocktail (80 ng/ml GM-CSF (PeproTech Inc., Rocky Hill, NJ) and 80 ng/ml IL-4 (PeproTech Inc.)) [21]. SF-DC Control + Serum medium was the SF-DC Control medium plus 10% fetal bovine serum (FBS, HyClone, Logan, UT). SF-DC Optimal medium was used in the following experimental designs and was RPMI 1640 medium supplemented with thirteen abovementioned serum substitutes plus 50 ng/ml GM-CSF, 50 ng/ml IL-4, 1.4 ng/ml IL-6 (PeproTech, Inc.) and 3 ng/ml M-CSF (PeproTech, Inc.).

### DC differentiation and maturation

For DC differentiation, CD14^+^ monocytes were initially seeded at 5 × 10^5^ cells/well (in 6-well plates, 1.5 mL medium per well, Corning, Glendale, AZ) in the indicated DC differentiation medium at 37 °C in a 5% CO_2_ humidified atmosphere. After 5 days of differentiation, 20 ng/mL TNF-α (PeproTech Inc.) or 1 μg/mL LPS (Sigma–Aldrich) was added to the medium to stimulate DC maturation for 48 h [40, 51–54]. After differentiation, cells were analyzed with the following assays at the indicated time points before (on Day 5) and after (on Day 7) stimulation with TNF-α or LPS.

### Experimental design of the SF-DC optimal medium formula - fractional factorial design and steepest ascent method

In this study, a two-level fractional factorial design and the steepest ascent method were combined to determine the optimal concentration of cytokines for DC differentiation from monocytes in the SF-DC Control medium. Factorial design data were regressed by Design Expert statistical software (Stat-Ease Inc., Minneapolis, MN) to obtain the polynomial. Statistical significance was determined by an *F* test, and the significance of the regression coefficients was analyzed by a *t* test. The polynomial takes the form of

4 $$\textrm{CD}4{0}^{+}\textrm{CD}20{9}^{+}\ \textrm{DCs}\ \left(\textrm{cells}/\textrm{mL}\right)={\upalpha}_0+\Sigma {\upalpha}_{\textrm{i}}{\textrm{x}}_{\textrm{i}}+\Sigma {\upalpha}_{\textrm{ij}}{\textrm{x}}_{\textrm{i}}{\textrm{x}}_{\textrm{j}}+\Sigma {\upalpha}_{\textrm{ij}}{\textrm{kx}}_{\textrm{i}}{\textrm{x}}_{\textrm{j}}{\textrm{x}}_{\textrm{k}}$$

Equation (4) is a simplified equation, where α represents the fitted constants, and x represents the coded variables for the tested cytokines. The constants αi, αij, and αijk correspond to the main effect, second-order interaction, and third-order interaction terms, respectively. We considered statistically significant main or interaction terms (*p* < 0.05) and neglected insignificant higher-order terms. Each positive constant in the equation can be used to screen the effective factors and provide information to construct the steepest ascent path to optimize the cytokine concentration for DC generation.

Strategies for the development of the SF-DC Optimal medium were as follows: (1) screen the effective cytokines from IL-1β, IL-2, IL-3, IL-6, IL-7, IL-12p70, IL-15, IL-16, IL-17A, Flt3-ligand, stem cell factor (SCF), hepatocyte growth factor (HGF), transforming growth factor-β (TGF-β), interferon-β (IFN-β) and M-CSF by factorial design in the SF-DC Control medium; (2) optimize the concentration of each effective cytokine (including IL-6 and M-CSF) by the steepest ascent path in the SF-DC Control medium; and (3) compare cell performance in the SF-DC Optimal medium with that in the SF-DC Control medium and SF-DC Control + Serum medium.

### Flow cytometry assay

Before and after differentiation, the cells were collected, washed and resuspended in Dulbecco’s phosphate-buffered saline (D-PBS, HyClone). To analyze surface antigens, anti-CD1a (BD Biosciences, San Jose, CA), anti-CD11c (BD Biosciences), anti-CD14 (Miltenyi Biotec Gmbh), anti-CD40 (BD Biosciences), anti-CD80 (Miltenyi Biotec Gmbh), anti-CD83 (Miltenyi Biotec Gmbh), anti-CD86 (Miltenyi Biotec Gmbh) and anti-CD209 (BD Biosciences) fluorescence monoclonal antibodies were used. Matched labeled isotypes were used as controls. The labeled cells were analyzed using flow cytometry (Accuri C6, BD Biosciences). The screening criterion of monocyte-derived DCs was defined as CD40^+^CD209^+^ cells in this study [55, 56].

### Cell morphology observation: scanning electron microscopy assay

The morphology of the differentiated cells was observed using scanning electron microscopy (SEM). After differentiation, the differentiated cells were harvested and transferred to a porous membrane for culture. Then, the cells were fixed with 4% glutaraldehyde (Sigma) and dehydrated stepwise with mixtures of ethanol and water that were progressively richer in alcohol. Finally, the cells on the porous membrane were sputter coated with gold for 100 s and observed under an SEM operated at 10 kV.

### Endocytosis analysis

After differentiation at the indicated time points, the differentiated cells were washed and seeded in 24-well plates at a final concentration of 2 × 10^5^ cells/ml in 1 mL/well. Dextran-fluorescein isothiocyanate (dextran-FITC, 1 mg/mL, Sigma–Aldrich) [57–59] or 1 mg/mL fluorescent latex beads (Sigma–Aldrich) [60] was added and incubated with cells in RPMI medium for 1 h at 37 °C in a 5% CO_2_ humidified atmosphere. Then, the cells were washed and stained with CD209-PE. The endocytosis ability was determined by the CD209^+^ cells that expressed the fluorescence of dextran-FITC or latex beads using flow cytometry.

### Cytokine secretion

To determine the cytokine secretion of the differentiated cells, the conditioned medium at the indicated time points was collected, and the secreted amounts of IL-1β, IL-8, IL-10 and IL-12p70 were analyzed by a human inflammatory cytokine cytometric bead array kit (BD Biosciences) using a flow cytometer according to the manufacturer’s protocol [61, 62].

### Mixed Leukocyte Reaction (MLR)

To test the allogeneic stimulatory activity of differentiated DCs, the differentiated cells were collected at the indicated time points and purified with anti-CD209 magnetic microbeads and a Vario-MACS Separator. The isolated CD209^+^ DCs that served as effector cells were preinactivated with 50 mg/mL mitomycin-C (Sigma–Aldrich) for 3 h to completely stop DC proliferation. CD3^+^ T cells were isolated from MNCs using CD3 microbeads (Miltenyi Biotec Gmbh) and the VarioMACS Separator. Then, CD3^+^ T cells were stained with carboxyfluorescein succinimidyl ester (CFSE, Sigma–Aldrich) to serve as the target cells. CD209^+^ DCs were cocultured with allogenic CFSE-stained CD3^+^ T cells at a ratio of 1:2 in RPMI medium supplemented with 20% FBS for 4 days at 37 °C in a 5% CO_2_ humidified atmosphere. Before coculturing, the mitomycin-C treated DCs were washed by D-PBS three times to completely remove mitomycin-C. After coculturing, total cells were harvested, and the percentage of proliferating CD3^+^ T cells in total T cells was determined using flow cytometry [63].

### Statistical analysis

All experimental results were from at least three independent experiments and are shown as the mean ± standard error. *P* < 0.05, *P* < 0.01 and *P* < 0.005 were considered to indicate statistical significance using the paired samples *t* test and are represented by *, ** and ***, respectively.

## Data Availability

The datasets used and/or analyzed during the current study are available from the corresponding author on reasonable request.

## Abbreviations

APCs: Antigen-presenting cells
CFSE: Carboxyfluorescein succinimidyl ester
DCs: Dendritic cells
Flt3-ligand: fms-like tyrosine kinase receptor 3 ligand
GM-CSF: Granulocyte-macrophage colony-stimulating factor
HGF: Hepatocyte growth factor
IFN: Interferon
IL: Interleukin
LPS: Lipopolysaccharide
M-CSF: Macrophage colony-stimulating factor
mDC: Monocyte-derived DC
MFI: Mean fluorescence intensity
MLR: Mixed lymphocyte reaction
SCF: Stem cell factor
SF-DC Optimal: Optimal serum-free DC differentiation medium
TGF: Transforming growth factor
TNF: Tumor necrosis factor
